# Plant-derived extracellular vesicles: a novel nanomedicine approach with advantages and challenges

**DOI:** 10.1186/s12964-022-00889-1

**Published:** 2022-05-23

**Authors:** Mohadeseh Nemati, Bipin Singh, Rakeeb Ahmad Mir, Mahdieh Nemati, Azadeh Babaei, Mahdi Ahmadi, Yousef Rasmi, Afsaneh Gholinejad Golezani, Jafar Rezaie

**Affiliations:** 1 Department of Biochemistry, School of Medicine, Urmia University of Medical Sciences, Urmia, Iran; 2 Department of Biotechnology, School of Engineering and Applied Sciences, Bennett University, Greater Noida, Uttar Pradesh 201310 India; 3 Department of Biotechnology, School of Bio-Sciences and Biotechnology Baba Ghulam, Shah Badshah University, Rajouri, Jammu & Kashmir 185234 India; 4 Department of Medical Nanotechnology, Faculty of Advanced Medical Science, Tabriz University of Medical Sciences, Tabriz, Iran; 5 Department of Anatomical Sciences, Faculty of Medical Science, Tarbiat Modares University, Tehran, Iran; 6 Drug Applied Research Center, Tabriz University of Medical Sciences, Tabriz, Iran; 7 Cellular and Molecular Research Center, Urmia University of Medical Sciences, Urmia, Iran; 8 Solid Tumor Research Center, Cellular and Molecular Medicine Institute, Urmia University of Medical Sciences, Shafa St, Ershad Blvd., P.O. Box: 1138, Urmia, 57147 Iran

**Keywords:** Extracellular vesicles, Exosomes, Plant-derived EVs, Biomedicine

## Abstract

**Background:**

Many eukaryote cells produce membrane-enclosed extracellular vesicles (EVs) to establish cell-to-cell communication. Plant-derived EVs (P-EVs) contain proteins, RNAs, lipids, and other metabolites that can be isolated from the juice, the flesh, and roots of many species.

**Methods:**

In the present review study, we studied numerous articles over the past two decades published on the role of P-EVs in plant physiology as well as on the application of these vesicles in different diseases.

**Results:**

Different types of EVs have been identified in plants that have multiple functions including reorganization of cell structure, development, facilitating crosstalk between plants and fungi, plant immunity, defense against pathogens. Purified from several edible species, these EVs are more biocompatible, biodegradable, and extremely available from many plants, making them useful for cell-free therapy. Emerging evidence of clinical and preclinical studies suggest that P-EVs have numerous benefits over conventional synthetic carriers, opening novel frontiers for the novel drug-delivery system. Exciting new opportunities, including designing drug-loaded P-EVs to improve the drug-delivery systems, are already being examined, however clinical translation of P-EVs-based therapies faces challenges.

**Conclusion:**

P-EVs hold great promise for clinical application in the treatment of different diseases. In addition, despite enthusiastic results, further scrutiny should focus on unravelling the detailed mechanism behind P-EVs biogenesis and trafficking as well as their therapeutic applications.

**Video Abstract**

**Supplementary Information:**

The online version contains supplementary material available at 10.1186/s12964-022-00889-1.

## Background

Extracellular vesicles (EVs) are a family of lipoprotein structures released by eukaryotic cells heterogeneously in origin, size, and content [1]. Three subclasses of EVs have been characterized, including exosomes are forming from the multivesicular bodies (MVBs), microvesicles derived from the cellular membrane, and apoptotic bodies’ originating from apoptotic cells [2, 3]. EVs are present in several biofluids and exchange bio-information between cells, representing another mechanism of intercellular communication [2, 3]. These distinctive biomolecule-decorated vesicles have been suggested to enclose the specific markers needed to discover their target both nearby and at distant locations [4]. Scientists in this field have focused on extracting EVs from animal cells and biological fluids to study their morphological and functional characteristics [5, 6]. A growing body of evidence shows that plant cells release EVs (P-EVs) containing bioactive molecules that show multiple functions. Previous studies have reported the presence of exosome-like nanoparticles (ELNs) in fruits, plants, and most recently in fungi [7]. Nanoparticles derived from edible plants such as grapes and grapefruit [8, 9], ginger [10] and carrots [11] have anti-inflammatory properties [12]. Tremendous therapeutic applications of P-EVs, such as anti-cancerous and anti-inflammatory effects, role in cell–cell communication and role in regulating intestinal homeostasis validate their warranting attention in the field of biomedicine [13]. Besides, P-EVs can successfully deliver exogenous and endogenous agents to animal cells in the majority of organs [14]. The simplicity of transport to animal cells and lack of cytotoxicity provides a good opportunity to utilize the P-EVs as agents to deliver drugs for therapeutic use [15]. From a drug delivery viewpoint, these vesicles are analogous to liposomes, known that both are phospholipid structures. However, EVs are natural and have cell origins that make them ideal carriers for delivering the therapeutic agent to specific sites. In addition, unlike some synthetic liposomes used in delivery, EVs are more biocompatible and safer and can genetically be modified and loaded with various exogenous agents [16, 17]. The current review was aimed to discuss the recent knowledge about P-EVs kinetics, types and their biochemical components. Moreover, this review has focused on P-EVs regarding therapeutic potential, advantages, and challenge.

## Therapeutic effects of plants

In this section, we describe the therapeutic effects of plants that their parts or EVs frequently used by researchers. Therapeutic effects of citrus fruits, the genus *Citrus* of the family Rutaceae, are frequently examined by researchers [18]. The flavanone hesperidin in citrus fruits, possess many pharmacological properties. Hesperidin have hepatoprotective properties that are induced via different mechanisms such as increasing the activity of heme oxygenase 1, nuclear factor-like 2/antioxidant response element and enzymatic and non-enzymatic antioxidants and reduction in the levels of high-mobility group box 1 protein, C-reactive protein, an inhibitor of kappa B protein-alpha and matrix metalloproteinase-9 [19]. Using mouse 3T3-L1 preadipocyte cells and human colon cancer HT-29 cells, Hirata et al. showed that the components from Citrus fruits have potential drugs for anti-corpulence and anticancer [20]**.** The major furanocoumarins (a specific group of secondary metabolites) found in grapefruits (Citrus paradisi) include 60,70-dihydroxybergamottin, bergamottin, and epoxybergamottin. The grapefruit furanocoumarins exhibit different biological activities such as anti-inflammatory, -cancer, and -oxidative activities and bone health promotion both in vitro and in vivo [21]. Grape seed extract (GSE) is represented by phenolic acids and polyphenols. GSE have anti-cancer activity via an increase in reactive oxygen species [22]. In addition, the consumption of grapes and grape products can decrease risk factors associated with cancer, cardiovascular health, age-related cognitive, and neurodegenerative disease. These effects are often attributed to the function of flavonoid compounds found in grapes, antioxidant activity, and increasing nitric oxide production [23]. Ginger, which belongs to the *Zingiberaceae* family, is a flowering plant that originated in Southeast Asia and it is among the healthiest spices on the planet [24]. Traditionally, ginger is used as a spice medicine to treat nausea and other gastrointestinal problems such as colic, bloating, diarrhea, and indigestion [25, 26]. This plant contains gingerol, which has powerful medicinal properties [27]. Gingerol has great anti- antioxidant and inflammatory properties, according to study [27]. For example, it may inhibit oxidative stress, which is the result of having an extra amount of free radicals in the body [28]. Antioxidants and other compounds in ginger root may aid inhibit or pleasure arthritis, inflammation, and various types of infection. Ginger may also decrease the risk of cancer and diabetes [27, 29]. Carrot (*Daucus carota*) is another plant that researchers have studied its medical effects [30, 31]. This plant contains loads of life-extending carotenes and is a great source of vitamin A. carrot juice also has the magical antioxidant glutathione, which defends against free radical harm, it shows powerful anti-inflammatory properties, serving to relieve the symptoms of rheumatism and arthritis [30, 31]. A study using in vivo model showed that the carrot juice has low concentrations of carotenoids that may be responsible for the anti-inflammatory and antioxidant effects to recover glucose tolerance, cardiovascular, and hepatic function [32].

## Types of P-EVs

**Fig. 1:**
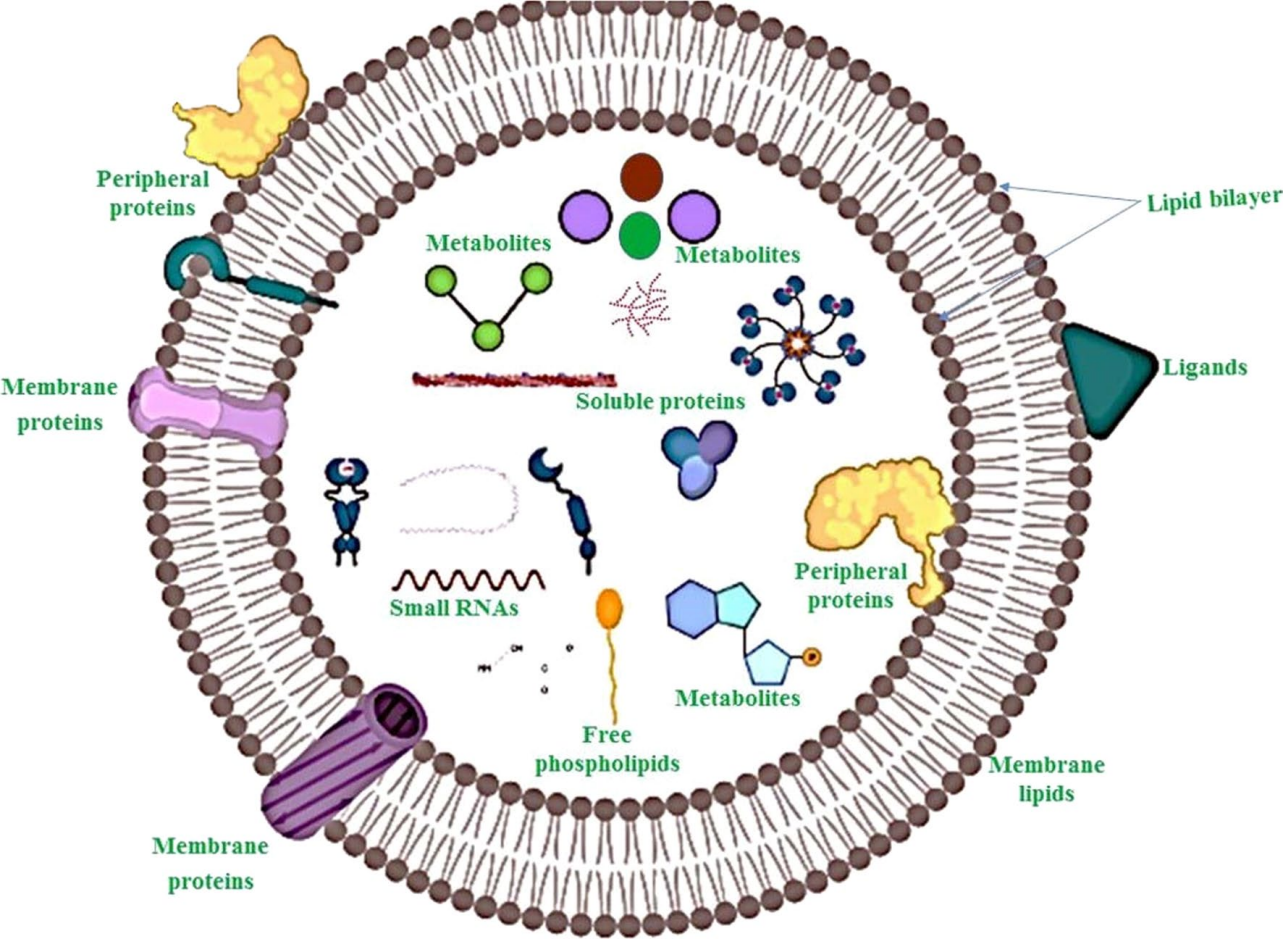
Representative figure showing the overview structure and generalized composition of plant-derived extracellular vesicles (P-EVs). P-EVs contain various biomolecules in their lumen and surface

**Fig. 2:**
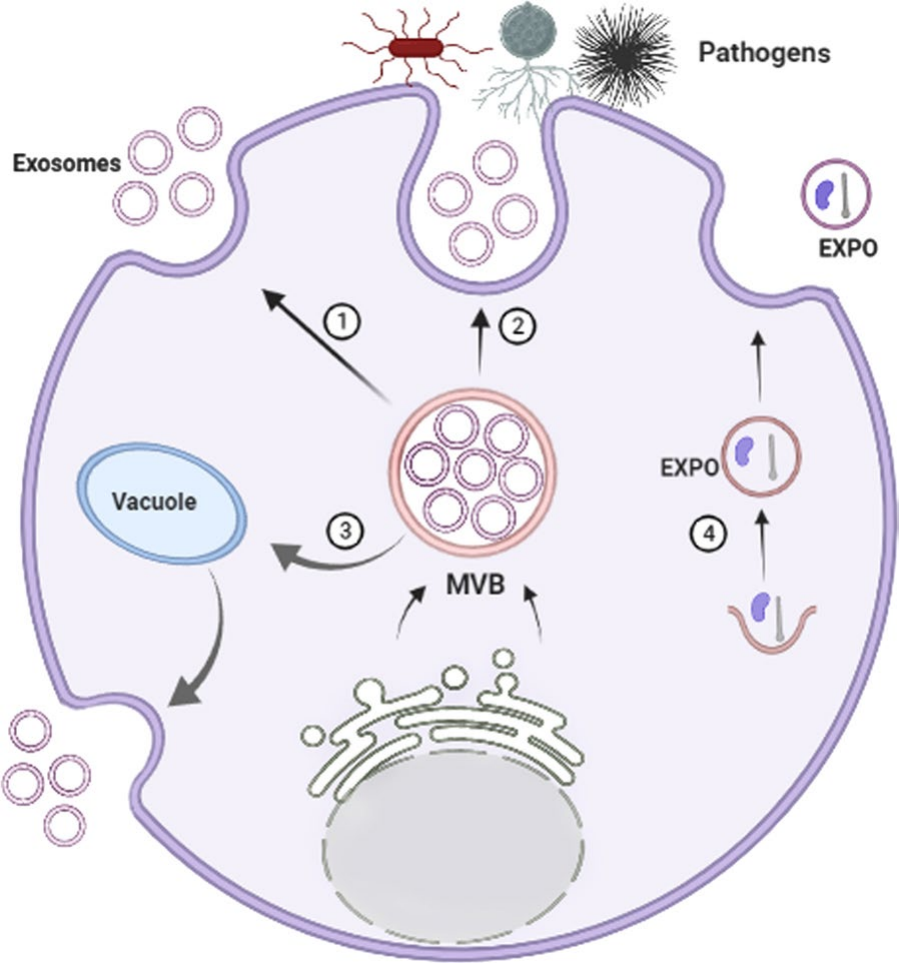
Schematic representation of the biological composition of P-EVs. These vesicles contain many bioactive molecules such as proteins, nucleic acid, and lipid that are not only cell structure molecules but also some have a cell-specific origin

EVs have been identified from a variety of cellular origins, such as mammalian cells, plant cells, and even bacteria [33]. P-EVs, similar to mammalian cells EVs, are membranous vesicles released by the plant cells into the extracellular space with key roles in intercellular communication [34, 35]. Research on edible plant vesicles has shown that P-EVs may be isolated from different parts of plants, including juice [36], flesh or roots [15], seeds [37], and dried plant materials [33]. They can be characterized by either density, size, conditions/cells of origin, or biochemical compositions [38, 39]. Although the cell wall of plant cells may prevent the formation and functions of P-EVs, however, there is much evidence that plants can produce EVs intending to mediate a wide range of cellular and physiological functions [40, 41]. The probable EVs in plants include MVBs, EXPO (exocyst-positive organelle), Penetration 1 (Pen1)-positive EVs, vacuoles, and autophagosomes [34, 35, 42]. Similar to EVs of animal cells, P-EVs contain diverse biomolecules such as proteins, lipids, RNAs, and metabolites (Fig. 1). MVBs were identified in plant cells in 1967 before their detection in mammalian cells [43]. It was said for the first time, distinct plants release exosome-like vesicles into the extracellular space by fusion of MVBs with the plasma membrane [43]. It would seem plant cells use a similar exosomes biogenesis pathway as animal cells use (Fig. 2). About half of proteins of EVs derived from *Citrus limon* belong to exosome-specific functional groups regardless of cellular origin or overlap with mammalian exosome proteins. These exosomes have been abundantly found in plant juices [44]. Two of the Arabidopsis tetraspanin (Tets)-like genes including, Tet8 and Tet9, are specifically identified in fungal pathogen *Botrytis cinerea* infection [45], which are co-localized with Arabidopsis MVB- marker Rab5-like GTPase ARA6 inside the cell and EVs at fungal infection sites [45]. It is also reported that the tet8 single mutant or the tet8 tet9 double mutant led to low secretion of EVs and sRNAs, and high susceptibility to *B. cinerea* infection [45, 46]. In addition, over fourfold in tet8 total leaf extracts decreases the amount of an EVs-enriched lipid, glycosyl inositol phosphoramides (GIPCs) [47]. Therefore, Tet8 may facilitate the EVs production in relation with GIPCs that result in defense against infections [48]. Tet8 is structurally alike CD63, an exosomal marker in animal cells. Seemingly, Tet8-positive EVs could be considered as plant exosomes, with a size 60–150 nm, which are produced upon pathological environments [49]. Whether there are different types of MVBs inside a specific plant cell or not, is a principle issue that should be considered in future studies.

Another type of P-EVs is EXPO [34]. Its double-membrane nature has been shown by microscopic analysis, which combines with the plasma membrane and produces another class of extracellular single-membrane-bound EVs [34] (Fig. 2). The EXPO-derived EVs, characterized by components of the exocyst complex Exo70E2 [34]. The size of EXPO-derived EVs is between 200 and 500 nm in diameter, which is larger than exosomes [34, 49]. The size of P-EVs was larger or similar to than exosomes of an animal cell and was similar to that observed for EVs derived from Arabidopsis rosette (50–300 nm) and sunflower apoplastic fluids (50–200 nm)[50]. Different laboratories purified different types of EVs from various plants, which are listed in Table 1.

**Table 1:** Characterization and drug-delivery application of P-EVs

Plant	Size (nm)	EVs morphology	Cell/disease model	Therapeutic agents	Administration route	References
Blueberries	Two EVs types	Spherical or oval	–	–	–	[51]
Oranges	Two EVs types	Spherical or oval	–	–	–	[51]
Carrots	80–200 and 700–1500	Cup-shaped or spherical	Intestinal homeostasis	–	Oral	[11]
Coconut	EVs from milk 30 EVs from water 13	Spherical	–	–	–	[52]
Watermelons	Two EVs types	Spherical or oval	–	–	–	[51]
Sunflower seeds	50–200	Spherical	–	–	–	[37]
Pears	Two EVs types	Spherical or oval	–	–	–	[51]
Soybean	Two EVs types	Spherical or oval	–	–	–	[51]
Tomatoes	100–1000	Spherical or oval	–	–	–	[51]
Arabidopsis leaves	50–300	spherical	–	–	–	[53]
Ginger	100–1000	Cup-shaped or spherical	Intestinal homeostasis	–	Oral	[11]
	189–232	Spherical	Ulcerative colitis	siRNA-CD98	Oral	[54]
	188	Spherical or *GiNVs*	Colon cancer	Doxorubicin	Intravenously	[55]
	219–292	Cup-shaped or spherical	Colitis-related cancer and Inflammatory bowel disease	–	Oral	[55]
	100–1000	Spherical	Liver diseases	–	Oral	[56]
	Two EVs types	Spherical or oval	–	–	–	[51]
	100–600	–	Periodontitis	–	Oral cavity	[57]
	50–150	Spherical	Gut diseases		Oral	[58]
	120–150	Spherical	inhibit NLRP3 inflammasome activity (Alzheimer)	–	Oral	[10]
Grapes	30–200	Spherical	–	–	–	[8]
	200–800	Spherical	DSS induced colitis –	Oral	–	[9]
	500–1000	Cup-shaped or spherical		Intestinal homeostasis	Oral	[11]
Grape fruits	50–100 and 100–1000	Cup-shaped or spherical	Intestinal homeostasis	–	Oral	[11]
	105–400	Cup-shaped or spherical	DSS-induced colitis	Methotrexate	Oral	[59]
	About 200	spherical	DSS-induced colitis Inflammation	Curcumin and Doxorubicin	Intravenously	[60]
	50–800	Cup-shaped or spherical	Cancer	PTX Folic acid	Intravenously	[15]
	72.5–102.5	Spherical	Brain tumor	miR-17	Intranasal	[61]
	110–120	- *GrpfNVs*	Liver cancer	siRNA IL-12 and miR-18a	Intravenously	[62]
	Two EVs types	Spherical or oval	–	–	–	[51]

Another class of P-EVs is vesicles that are formed from vacuoles or/and autophagosomes (Fig. 2). Cui et al. [63] showed that small vacuoles contain intraluminal vesicles (ILVs) that attribute to the fusion of MVBs with them. Recent developments in P-EVs have reported that specific types of MVBs may deliver ILVs to vacuoles through fusion with them where finally vacuoles fuse with the plasma membrane to release the remaining ILVs out of the cell [64] (Fig. 2). There is evidence that following a bacterial infection, plants recruit the vacuole to fuse with the plasma membrane, and consequently secrete vacuolar defense molecules into the extracellular space to combat the bacteria infection [64]. Therefore, a distinct type of vesicles may result from the fusion of vacuoles and the plasma membrane [64]. These EVs carry defense protein against bacteria to prevent their infection. Another type of P-EVs may result from autophagosomes, however, unlike animal cells, the pivotal role of autophagosomes in P-EVs biogenesis and secretion has not been extremely confirmed yet [64, 65].

## P-EVs function

As mentioned above, The P-EVs contribute to different processes such as intercellular communication [12], basal immunity, and response to environmental stress [53, 66]. For example, the secretion of cell wall-related proteins is essential for plant cells to the reorganization of the wall and maintenance cellular homeostasis under environmental stress [67]. In addition, due to the endosomal origin of MVBs, they are involved in the sequestration of damaged membranes and harmful substances resulting from oxidative microburst and internalization of nutrients from apoplasts. Therefore, because of the delivery of cargo to the extracellular space or central vacuole, the P-EVs play a critical role in cell proliferation and cell response to stress [13]. Due to the release of ELNs into the extracellular matrix and EVs into the apoplast, it has been suggested that secretion of exosomes and EXPO-derived vesicles are as unconventional protein secretion ways in plants [34, 68]. EXPO may have several roles in the re-localization of defence-related molecules during pathogen invasion and cell wall building [34, 69]. In addition, EXPO contains hydrolytic enzymes and defence protein that possibly play a significant role in the plant defence system [70]. P-EVs are highly stable in various environmental conditions and are resistant to degradation by digestive enzymes for their physicochemical structures [16]. For example, grapefruit-derived EVs were shown to be resistant to bile extract solution, pepsin, and intestinal pancreatin [59]. Interestingly, it was shown that the physicochemical features of ginger-derived EVs including surface and charge size, may change upon incubation with virtual stomach solution (pH 2) or virtual small intestine medium (pH 6.5)[56]. The P-EVs could cross through the intestinal barrier. Data from fluorescent membrane dyes indicated a grape-EVs accumulation in the gut during the first 6 h post-gavage, and its decrease over 48 h [9], ginger-EVs, high retention of orally administered EVs in the non-starved mice colon rather than starved mice 12 h post-treatment [71]. In addition, P-EVs derived from many plants can also accumulate in a wide range of mammalian cells in vitro, such as, in intestinal stem cells [59], cancer cells [44], macrophages [11, 54], Caco-2BBE cells, colon-26 cells, and HT-29 cells [54, 71], dendritic cells [72], and primary hepatocytes [56]. There are four mechanisms by which EVs can interact with recipient cells including: (a) surface protein interaction (receptor-ligand interaction), triggering signal transduction in the target cell, (b) fusion, (c) endocytosis and (d), activation of an EVs-bound surface protein by proteases located in the extracellular matrix. In this regard, EVs derivatives may bind to respective receptors on target cells as a ligand [73]. The entry of P-EVs may change according to the state of the recipient cells. For example, ginger-derived EVs were internalized at a higher rate by the mice liver fed with an ethanol diet (induces fat accumulation and extensive steatosis in the liver), compared to mice fed with a regular diet without liver damage [56].

In mammalian cells, vesicle uptake is often mediated by endocytosis rather than by the fusion of vesicles and the plasma membrane [74]. However, uptake of EVs by plant cells is complex and remains unclear. It can be suggested that plant-vesicle uptake is highly cell type-specific [13]. For instance, uptake of garlic-derived nanovesicles by liver cells perform via interaction among the transmembrane glycoprotein heterodimer CD98 and a mannose-binding lectin [75], but uptake of grape-derived EVs is dependent on both clathrin-dependent and micropinocytosis pathways for entry into macrophages [59].

## Biological composition and function of P-EVs

**Fig. 3:**
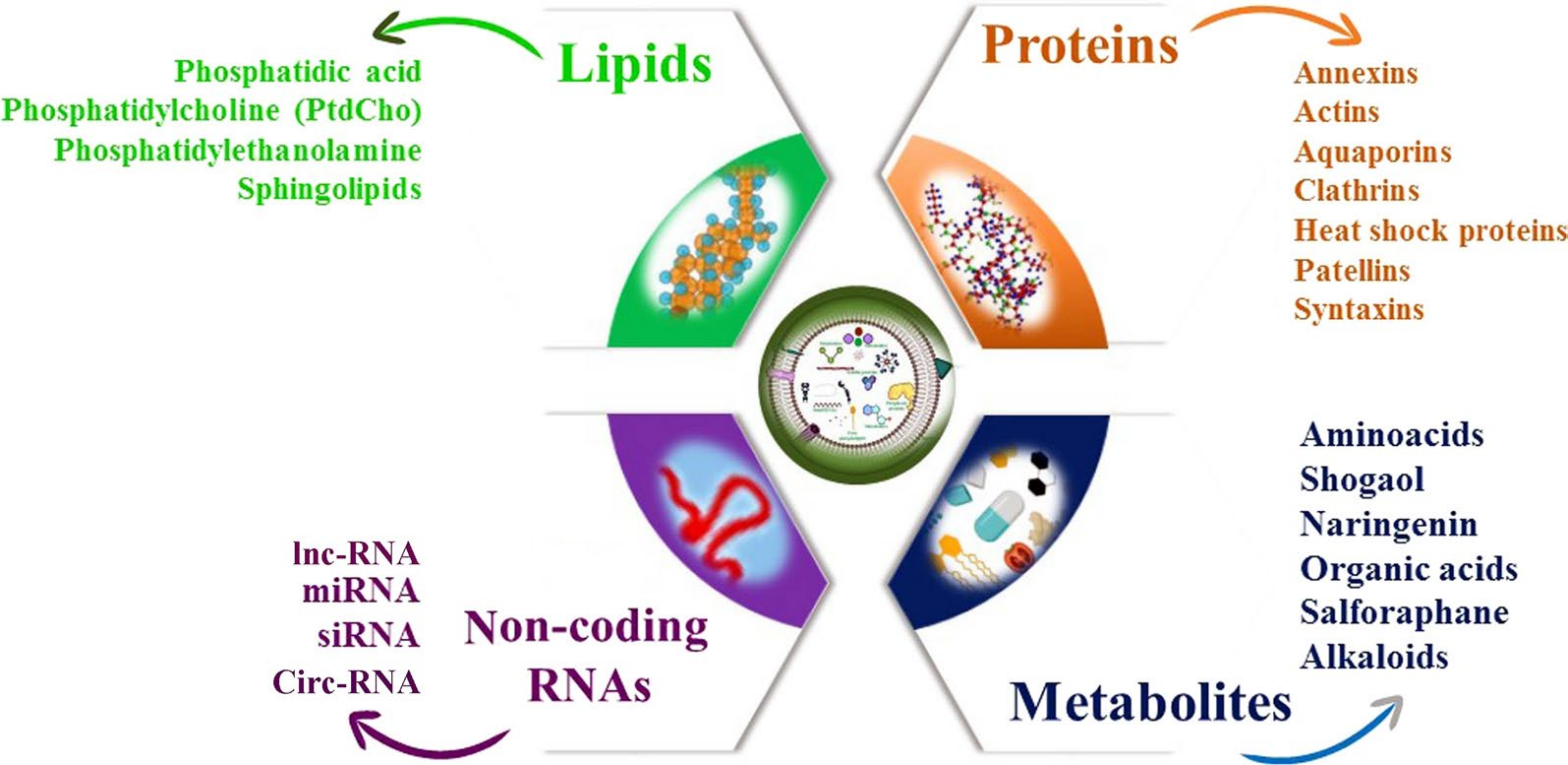
Extracellular cellular (EVs) biogenesis in plant cells. Exosomes biogenesis and trafficking in plant cells mechanistically are similar to those of animal cells. Exosomes are generated from multivesicular bodies (MVB) inside the cell with Tet8 marker, which is an alternative to CD63 protein of animal exosomes. MVBs may fuse with the plasma membrane to release distinct types of exosomes for intercellular communication (1) or defense against various pathogens infection (2). Furthermore, MVBs may fuse with the vacuoles to form MVBs-Vacuole hydride vesicles that finally fuse with the plasma membrane and exosomes are released into extracellular space (3). It is unknown that whether plant cells contain different types of MVBs with specific exosomes or not. Another subtype of P-EVs has known as the EXPO that formed independently of the MVBs pathway (4)

Similar to mammalians EVs, P-EVs contain many kinds of biomolecules that contribute to regulating the fate and morphology of recipient cells [1] (Fig. 3). However, P-EVs are not well explored compared to the vesicles derived from animal cells; hence a comprehensive understanding of the biomolecular cargo composition of P-EVs is still absent [70, 76]. The type of cargo of P-EVs is influenced by the condition under which they are secreted or synthesized [70, 76]. In this section, we focus on the biological compositions of P-EVs.

### Proteins

P-EVs isolated from different plant sources suggest the presence of a highly diverse range of proteins from different classes. These include various proteins involved in environmental and pathogen stress, metabolic signaling, cell-wall expansion, cytoskeleton formation, cellular transport, and secretory pathways. We describe some of these crucial proteins found in P-EVs [77, 78].

#### Annexins

Proteins from the Annexin family have been identified in EVs extracted from Citrus juice and apoplastic fluids, Sunflower seed, and Arabidopsis leaves. The presence of Annexins has been related to biotic and abiotic stress responses in plants [53].

#### Heat shock proteins

Heat Shock Proteins (HSP60, 70, 80, and 90) have been reported from EVs extracted from Citrus fruit, sunflower, Olive, Grapefruit, Grapes, and tomatoes. Many studies have reported that the presence of a wide range of HSPs deal with the both biotic and abiotic stress [44, 53, 79, 80].

#### Aquaporins

Proteins belonging to the aquaporin family have been reported from P-EVs isolated from citrus grape, broccoli, and Arabidopsis. The presence of aquaporins has been correlated with the stability of the plasma membrane in P-EVs and their water permeability [79, 81].

#### Proteins involved in cell-wall remodeling

P-EVs derived from pollens have been found to contain pectin methylesterase that is involved in cell wall expansion [82].

#### Actins

Actins are carried by P-EVs derived from ginger and Arabidopsis. The presence of actins has also been reported in EVs derived from pollen and citrus. The presence of actin hints at many possible active processes in P-EVs, such as cell division, cell expansion, organelle movement, and vesicle trafficking [33, 78].

#### Patellins

Various members of Patellins (Patellin 1, 2, and 3) have been found in EVs of Arabidopsis and citrus. As per the information provided at Uniprot, Patellins are carrier proteins that may be involved in membrane-trafficking events associated with cell plate formation during cytokinesis. It binds to some hydrophobic molecules and promotes their transfer between the different cellular sites. Thus, the presence of these class proteins in P-EVs is important for functionality in plants [79, 83].

#### Syntaxins

P-EVs isolated from Arabidopsis and citrus have also been found to contain syntaxins that are considered as key proteins in vesicle trafficking, fusion, and exocytosis and are involved in various important secretory pathways [79, 84].

#### Clathrin heavy chain

Members of the clathrin heavy chain family (Clathrin 1, 2) have been found in EVs extracted from Arabidopsis, citrus, and sunflower. These proteins belong to the endosomal pathway that is known to play a vital role in the formation of clathrin-coated vesicles and endocytosis [79, 84].

#### RAS-related proteins

Various members of RAS-related proteins (RabA2a, RabA2b, RabB1c, Rab7, Rab2A, 5C, 6A, 7A, 8A, 11A, and 18) have been reported in EVs derived from primarily from Arabidopsis and citrus. Most commonly, these proteins are present in P-EVs to aid in cargo sorting, vesicle transport, and secretion [44, 53, 79].

### Lipids

The lipid composition of P-EVs is different from that of EVs extracted from animal cells. However, the study of the lipid composition of P-EVs has become attractive since it has been shown that they can interact with and internalize by animal cells [85]. Therefore, the composition of lipids has a direct impact on the biological function of P-EVs [47]. The major lipid constituents including Phosphatidic Acid (PA), Phosphatidylethanolamine (PE), and Phosphatidylcholine (PC) have been found in P-EVs [84, 86].

PA has been reported in P-EVs extracted from sunflower apoplastic fluid, grape, orange, and ginger [84]. The role of PA has been shown in mitogenesis, membrane fusion, and fission processes [87]. PE and PC were reported to be present in P-EVs from grapefruit, orange, and ginger [56]. The structural and chemical features of PA influence membrane curvature. PA acts as a signaling lipid and facilitates the recruitment of appropriate cytosolic proteins to the membrane [88]. In addition, this molecule participates in vesicle formation by promoting membrane curvature and recruiting the appropriate proteins [89]. Therefore, polyunsaturated fatty acids in PA, PE and PC may help the EVs to reduce rigidity, which might help during the process of endocytosis that requires membrane bending and deformation [89]. The polar head group in PE creates a more viscous lipid membrane compared to PC [90]. Therefore, P-EVs having PE over PC may have a context-dependent functional advantage [90]. PC also plays a role in membrane-mediated cell signaling [88]. Thus, the presence of a particular lipid type in P-EVs provides them with a specific functional advantage. PA and PC in ginger EVs play an important role in migration from the intestine and uptake by intestinal bacteria [10]. Thus, the presence of a specific lipid type in PDEVs can favorably modulate the intestinal microbiota [91, 92].

#### Sphingolipids

Sphingolipids are reported in EVs derived from Arabidopsis and Tobacco and may contribute to the dynamic nature of the P-EVs membrane [93]. Glycosylated inositol phosphoryl ceramides (GIPCs) are the predominant sphingolipids in plant tissues [93]. Although sphingolipids have a better-defined role in animal systems, they are central to many essential processes in plants, but not limited to pollen development, signal transduction, and in response to biotic and abiotic stress [94]. Therefore, a better understanding of the structure and composition of various lipids found in diverse P-EVs is desired [92, 94].

### Short non-coding RNAs

P-EVs have been shown to contain small non-coding RNAs such as micro RNAs (miRNAs) [76]. The miRNAs are present in most P-EVs, such as in ginger, grape, grapefruit, lemon, broccoli, and apple [58]. The functional role of miRNAs has been implicated in the therapeutic targeting of specific cells and interkingdom communications [95]. Using in vitro methods, miRNAs from P-EVs can regulate the expression of inflammatory cytokines and cancer-related genes [86, 91]. The role of miRNAs has also been implicated in targeting several genes in different bacteria in the host thus modulating the host microbiome. A total of 418 conserved miRNAs were identified from eleven edible fruits and vegetables based on in-silico prediction [96]. Most of these miRNAs were predicted to target human genes involved in immune response and cancer-related pathways [96]. It was reported that EVs of Arabidopsis contain short RNAs that could deliver to infection sites that are then taken up by the cells of fungus *Botrytis cinerea* [97]. Therefore, Arabidopsis has adapted cross-kingdom RNA interference-mediated by EVs as a part of its immune response to winning over the fungal infection. Unfortunately, identifying miRNAs from P-EVs is challenging because the pre-miRNAs sequences are not available or incomplete for specific plants [84, 86, 91, 98]. Thus, overall, miRNA from P-EVs possesses the promising potential to modulate gene expression in a particular pathological condition.

### Metabolites

The metabolic profile of P-EVs is highly variable because different plants produce a wide range of bioactive molecules involved in primary and secondary metabolism [84, 86, 91]. We highlight currently reported metabolites in P-EVs from important plant sources [84, 86, 91]. P-EVs isolated from broccoli have been found to contain sulforaphane. There have been few reports that suggest it has cancer-protective properties [99]. P-EVs from grapefruit contain naringenin, fructose, citric acid, glucose, sucrose myo-inositol, quinic acid, oxalic acid, glycolic acids, aucubin in addition to amino acids leucine and isoleucine [82, 91, 100]. Among these conventional metabolites, naringenin has been reported to possess anti-tumor properties [101]. P-EVs from Strawberry were found to be rich in ascorbic acid [102]. Apples derived EVs have flavonoids and furanocoumarins that are reported to be toxic against fungal species and insects [103]. The P-EVs isolated from Javanese ginger and turmeric carry curcuminoids, which is well-cited for their antioxidant properties [82]. P-EVs derived from tobacco contains alkaloid and phenolics and EVs from aconitituber are loaded with traces of aconitine, hypaconitine, and mesoaconitine that have toxic nature [100]. Thus, P-EVs is loaded with a wide variety of metabolite cargo based on their origin, thus possessing great potential for future therapeutic application.

## Therapeutic application of P-EVs

**Fig. 4:**
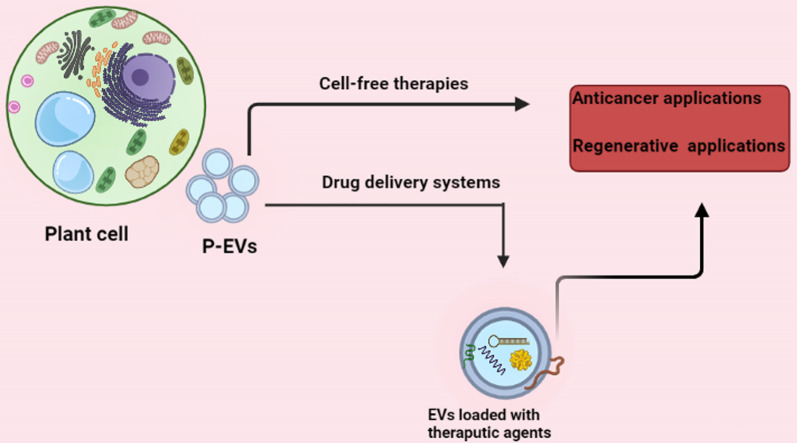
Potential therapeutic application of P-EVs in medicine. P-EVs represent therapeutic properties similar to their origin cells, therefore they may be useful for different pathologic occurrences, including cancer and degenerative diseases as cell-free therapy approaches. P-EVs have several advantageous properties that make them superior to EVs of animal cells in nanotechnology regarding the drug-delivery system

EVs derived from non-toxic plants can eliminate the problems associated with existing nanosized delivery systems due to their natural composition, origin, and opportunity to extract them in bulk from economical plant sources [85] (Fig. 4). P-EVs derived from edible plants are particularly very promising due to their abundant availability, biocompatibility, and biodegradability, therefore these EVs may be useful as a cell-free therapy for several diseases (Fig. 4 and Table 1).

For therapeutic progress, drug nanocarriers should be intensely evaluated on physicochemical characterization and communications within various biological environments [104]. While liposomes have been widely assessed, EVs showed the unique properties that make them superior for drug-delivery systems [105]. EVs released by plants cells can be used as drug carriers as offering advantageous properties, comprising low immunity, tissue-specific targeting, safety, large-scale production, favorite negative zeta potential values, and the ability to load many biomolecules [15]. P-EVs can be loaded with many therapeutic agents, including drugs, proteins, DNA vectors, and siRNAs, delivering them to target tissues (Table 1). In recent years, in the area of nanotechnology, EVs have attracted scientists’ attention due to their drug delivery potential, as these particles can deliver hydrophobic and hydrophilic therapeutic agents to target locations of disease [106] (Fig. 4). It was reported that particles purified from grapefruit showed high stability and can convey several agents such as curcumin, Zymosan A, and FA that were functionally active [15]. Wang et al. [60] used grapefruit-derived EVs to deliver doxorubicin (Dox) to the inflamed tumor site. These EVs were stable and capable of releasing Dox. Further scrutiny showed that these EVs could be loaded with various agents like chemotherapeutic agents drugs, and anti-inflammatory compounds such as curcumin. In vivo study using various mouse models of inflammation showed that the leukocyte-coated P-EVs promoted the efficiency of Dox delivering to inflammatory sites [60]. It could be concluded that P-EVs may be applicable as carrier tools for several drug delivery uses in preclinical experiments. However, more valuation in clinically related methods and straight, computable evaluation with liposome-based methods are essential to carefully measure the benefit/risk ratio. In this section, we describe the potential therapeutic application of P-EVs based on plant types. Table 1 has summarized the application of P-EVs in diseases models.

### Citrus fruits

Grapefruit is one of the plant food sources that has been studied due to its use in the delivery of nanomedicines. Wang et al. [60] showed that grapefruit-derived nanocarriers (GNV) were very effective in providing a variety of therapeutic agents, including drugs, DNA expression vectors, siRNA, and antibodies, using both in vitro cell culture and mouse. They also used GNVs as drug delivery system for delivering theaputic agents in different cells where they did not show any cytotoxicity or inducing an inflammatory cytokine response [15]. In another study, they showed that ginger-derived nanovesicles (GDNs) were selectively absorbed by intestinal macrophages and improved DSS-induced rat colitis [59]. They also confirmed that GDNs are biodegradable and stable over a wide range of pH values and suggested that they could be developed as a new oral drug delivery system. Accordingly, they introduced an anti-inflammatory drug (methotrexate, MTX) into GDNs and found that the encapsulated drug was less toxic than free MTX and showed significantly greater therapeutic effects against DSS-induced colitis in mice [59]. Their findings show that GDNs modulated the immune responses in the gut and maintained intestinal macrophage homeostasis [59]. In addition, in another study, authors used in vitro and in vivo experiments where they demonstrated that GDNs can specifically transfer therapeutic agents with folic acid, which in turn resulted in increasing targeting efficiency to tumor cells expressing folate receptors. The therapeutic potential of GDNs was further shown by inhibition of tumor growth in both CT26- and SW620-induced tumors in mice models [12]. The authors concluded that thesenano-vectors were less toxic than nanoparticles made from synthetic lipids and did not cross the placental barrier when injected intravenously into pregnant mice, suggesting that they could be useful for drug delivery [12].

Nanoparticles derived from edible plants (grapes, grapefruit, ginger and carrots) have anti-cancer properties in [9, 44, 59]. For instance, Raimondo and colleagues reported that lemon-derived EVs inhibited cell proliferation in three tumor cell lines including A549 (human lung cancer), LAMA84 (human chronic myeloid leukaemia), and SW480 (adenocarcinoma, Human colon) [44]. These vesicles inhibited the growth of the CML xenograft model in vivo, and these nanoparticles exert their anticancer activity by stimulating the apoptotic mechanism of TNF-related apoptosis-inducing ligand (TRAIL) as an alternative method for cancer detection [44]. Further studies from the same group showed that proteins belonging to the lipid metabolism pathway are modulated differently by the treatment of lemon-derived EVs in the colorectal adenocarcinoma cell line [107]. These nanoparticles acted as an antioxidant agent due to the content of small RNAs, citrate and vitamin C, based on an in vitro study using mesenchymal stromal cells (MSCs) [108]. It has also been reported that probiotics manipulated with lemon-derived EVs can inhibit Clostridium difficile through AhR-dependent and AhR-independent pathways. Treatment with these EVs increases I3Ald and I3LA levels while decreasing indole levels in the gut. I3Ald and I3LA act as AhR ligands and activate AhR, which in turn activates IL-22 expression, and IL-22 plays an important role in reducing the severity of many intestinal infections. These metabolic reactions increase the production of lactic acid in the gut, inhibit *C. difficil* growth, and reduce mortality and transmission [109].

### Ginger

Gingeris one of the most widely used natural products [21]. Ginger has many beneficial properties such as antioxidant [21], antibacterial [22], anti-inflammatory [13], anti-cancer [23], and regenerative potential for various diseases [25],, Zhang et al*.* isolated nanoparticles in large quantities from *Zingiber officinale* and identified ginger-derived nanoparticles (GDNVs) [71]. They found that GDNVs were effectively absorbed by intestinal cancer cells, and the modification achieved active targeting for colon-26 tumors in vivo by targeting folic acid (FA) ligand. Most importantly, GDNVs, made from ginger lipids, showed excellent biocompatibility, and Dox-loaded GDNVs successfully inhibited tumor growth in the colon xenograft tumor model [71]. Another study showed that intestinal macrophages and intestinal stem cells could uptake GDENs in the colon [55]. GDENs were able to maintain intestinal homeostasis. They increased the gene expression of both the anti-inflammatory cytokines HO-1 and IL-10, as well as the proinflammatory cytokines such as IL-6 and TNFα [55]. Using various mouse colitis models, authors demonstrated that GDNPs decreased acute colitis, increased intestinal repair, and inhibited chronic colitis and colitis-associated tumor [55].

GDENs have an anti-inflammatory effect by lowering the level of lipocalin-2, a biological marker of intestinal inflammation [110]. GDENs also increase the production of anti-inflammatory cytokines while decreasing the production of proinflammatory cytokines [110]. Also, through in vivo and in vitro assays, GDENs have been demonstrated to repair the mucous tissue of mice caused by colitis [55]. In addition, GDENs prevent the accumulation and activation of NLRP3 inflammation, the abnormal activity of which is associated with various diseases such as multiple sclerosis, atherosclerosis and type 2 diabetes [10]. Studies have also shown that GDENs contain small amounts of proteins and miRNAs, 124 of which can potentially regulate the expression of human genes, various types of lipids as well as biologically active compounds. The biologically active compounds of ginger, including 6-gingerol and 6-shogaol, exert antioxidant, anti-inflammatory and anti-cancer activities [55, 111–113]. In addition, GDENs in *Porphyromonas gingivalis*, the causative agent of gingivitis, enter the bacterium through the interaction between phosphatidic acid in the membrane of GDENs and protein 35 (HBP35) in the outer part of *P. gingivalis*, reducing its pathogenicity. It inhibits its ability to attach to and attack oral epithelial cells and prevents tooth decay caused by *P. gingivalis* [114]. Studies show that ginger has a hepatoprotective effect against ethanol, carbon tetrachloride and acetaminophen-induced hepatotoxicity [56]. In another study, Teng et al. [115] stated that GDENs are preferably consumed by Lactobacillaceae, while grapefruit exosomes are preferably consumed by Ruminococcaceae. They also found that the administration of GDENs to mice increased the intestinal microbial population of Lactobacillaceae and Bacteroidaceae and decreased the population of Clostridaceae [85]. Therefore, plant vesicles can alter the microbial composition of the gut, suggesting their potential use in the treatment of intestinal dysbiosis and related disease [85].

### Other plants

The use of herbal EVs will be excellent candidates for therapeutic agents because they can cross mammalian barriers without causing an inflammatory reaction or necrosis [55, 60] (Fig. 4). Wang et al. [15, 60] showed that nanoparticles from grapes naturally contain small RNAs, proteins, and lipids. Although these nanoparticles may not be identical to mammalian cell-derived exosomes, the structure and composition of grape nanoparticles are similar to those of mammalian-derived exosomes [9]. Grape-derived exosomal-like nanoparticles (GELNs) contain specific proteins, including aquaporin and HSP70 proteins, lipids enriched for PE and PA. In a study, using GELNs, it was shown that they have unique transport properties and biological functions [9, 116]. GELNs can penetrate the intestinal mucosal barrier and are captured by mouse intestinal stem cells, which in turn significantly induced intestinal stem cells via the Wnt/β-catenin pathway [116]. Oral administration of GELNs protects mice against colitis induced by sodium dextran sulfate (DSS) through induction of intestinal stem cells [9]. P-EVs can bind to many hydrophobic drugs such as curcumin due to their high lipid content [15]. For example, the therapeutic approach in chronic inflammatory diseases such as colitis is the systematic use of anti-inflammatory drugs that can have many side effects [117]. The plant-derived exosome may be useful for drug delivery systems that specifically deliver the drug to the inflammatory cells in the intestine, which could be a new therapeutic approach. In this regard, Wang et al. [59] showed that GELNs in the DSS-induced mouse colitis mainly absorbed macrophages and increased the anti-inflammatory capacity of intestinal macrophages. To increase their therapeutic effects, GDNs was used as a delivery system for anti-inflammatory and immunosuppressive methotrexate drug. Their results showed that methotrexate binding GDNs targeted macrophages in the lamina propria and ultimately reduced inflammatory cytokines such as TNF-α and IL-1β and decreased expression of the neutrophil chemokine KC [59]. These findings proposed that GDNs can act as immunomodulatory in the gut and preserved intestinal macrophage homeostasis, therefore can be developed for oral delivery of small molecule drugs used to minimize inflammatory responses in human disease [59]. In an in vitro study, blueberry-derived exosome-like nanoparticles (B-ELNs) were adsorbed by a human stabilized endothelial cell line (EA.hy926) in a dose-dependent manne. B-ELNs induced the production of TNF-α-induced reactive oxygen species (ROS), the loss of cell viability, and modulate the expression of TNF-α-induced genes. As a result, authors concluded that B-ELNs can serve as therapeutic carriers for bioactive compounds [7].

Common edible fungi contain ELNs, which is composed of lipids, RNAs, and proteins [114]. Among fungal-derived ELNs, those acting on shiitake fungi, S-ELNs, showed strong anti-inflammatory activity [114]. Seven fungi were commonly used to extract ELNs [118]. Among these mushroom-derived ELNs, only shiitake mushroom-derived ELNs (S-ELNs) substantially inhibited NLRP3 inflammasome activation by preventing inflammasome formation in primary macrophages [109]. In contrast, the other six fungi do not inhibit the production of NLRP3. S-ELNs also suppressed IL-6 secretion as well as protein and IL-1b gene mRNA levels [118]. Significantly, pretreatment with S-ELNs protected mice from acute GalN/LPS-induced acute liver damage [118]. As a result, S-ELNs are known as potent inhibitors of NLRP3 inflammation and have therapeutic effects in relieving the common disease of liver failure (FHF) in humans [114, 118].

## Clinical trials

According to the promising results from the effects of vesicles extracted from plants, several clinical trials were started to complete the results (Table 2). The use of plant exosomes in clinical trials began in 2012. However, complete results of clinical trials using plant exosomes have not been reported so far, and studies are in the early stages. As shown by Table 2, four types of plant-derived exosomes namely curcumin, ginger and grape have been used in clinical trial studies with the same supporter, the University of Louisville.

**Table 2:** P-EVs based clinical trials

ID no.	Phase	EVs source	Loaded agent	Condition	Administration	Status
NCT01294072	I	Plants	Curcumin	Colon cancer	Oral *Tablets*	Active, not recruiting
NCT03493984	Preliminary Clinical Trial	Ginger Aloe	Not determined	Insulin-related conditions Chronic inflammation in patients	–	Recruiting
NCT01668849	I	Grapes	Not determined	Oral Mucositis	*Dietary Supplement* oral, daily for 35 days	Active, not recruiting

The clinical trial with ID number: NCT04879810 investigated the effect of ginger and curcumin exosome on inflammatory bowel disease. The anti-inflammatory effect of exosomes is evaluated after 28 days using a biopsy, a blood sample, and assessing the quality of life. This clinical trial was performed randomly and in parallel on 35 participants and the status of this study is recruitment. In another clinical trial (NCT01668849), grape-derived exosomes were used as an anti-inflammatory agent to reduce oral mucositis in patients with head and neck cancer who underwent chemotherapy. Evaluations are performed after 6–7 weeks of treatment. Clinical Trials (No. NCT01294072) were run in 2011 that used curcumin conjugated with plant exosomes for more effective release of curcumin in the intestinal. The effect of curcumin conjugated plant exosomes on malignant and normal clone cells as well as its effect on the immune system in people with colon cancer is investigated. In this pilot study, the intervention group consisting of people who received curcumin, curcumin combined with plant exosomes and people who did not have any intervention were included in this study. This study was designed to compare the delivery effect of curcumin by plant exosomes with oral tablets and in recruitment status.

## Advantages and challenges

The interdisciplinary significance of EVs-based researches has fascinated hopeful interests, and the EVs methodical platforms for their regenerative properties, anti-diseases, drug-delivery, and diagnostic prospects have expressively advanced [104]. The current technologies and methodology formerly considered for mammalian EVs may also be functional for the isolation and characterization of P-EVs [70]. EVs-based therapies have many advantages with different challenges. For instance, EVs from animal cells show helpful properties regarding treatment of many diseases, however, one of the main challenges in this field is the challenge of whether and how the appropriate amounts of human EVs might be produced in vitro or purified from biological fluids. Indeed, the EVs produce per unit of initial substantial will influence the final making cost and clinical applications [119]. Therefore, the selection of alternative sources of EVs is fundamental. Furthermore, P-EVs have a natural origin and the opportunity of separating from large volumes, which signify the main advantages for their bio-plication [120]. However, there are challenges in evaluating the results and conclusions from the consequences remain undefinitive, due to different approaches being engaged for EVs isolation and characterization by different studies. Some of the studies did not include ISEV guidelines for the EVs characterization and their function, because those experiments had been done before the 2014 and 2018 statement of ISEV guidelines of minimal experimental necessities for EVs-based studies [38, 121]. For example, some laboratories did not separate any EVs from samples and reported a variety of size ranges for EVs (Table 1) or different laboratories used different methods for isolation (such as ultracentrifuge, commercial kits, etc.) and characterization (electron microscopies, flow cytometry, DLS, etc.) of EVs. Thus, systematic analysis of the efficiency and safety of P-EVs needs to resolve their individuality and purity. Another problem is that clear evidence of P-EVs characteristics including surface markers, densities, and sizes are still infrequent [120]. Biogenesis pathways are also not well known exactly, although, for instance, the term ‘MVBs/vesicles’ has been associated with putative exosomes and presented accumulating in the apoplast at the place of fungal penetration in barley leaves [122]. EVs have also been found in the apoplast of many grapes [8]. It seems that due to the current lack of knowledge, it is still difficult to classify P-EVs with the current nomenclature used for animal EVs. Thus, the P-EVs literature shows a confusing array of terms, including exosome-like vesicles, exosomes, microvesicles, and nanovesicles.

Nevertheless, in recent years, there has been an increasing interest in using EVs as a novel agent in nanomedicine as follows [70, 104, 123]: (1) Using EVs as a drug-delivery system. A growing body of experiments has shown the unique properties of EVs that make them ideal for delivering anticancer agents (biological molecules and drugs) to cancer cells. Therefore, researchers have endeavoured to load EVs with therapeutic agents by two methods well-known as direct loading mechanism and indirect loading mechanism (to further study, see review article [124]). For example, grapefruit-derived EVs were loaded with Dox to deliver it to the inflamed cancer site [60]. Therapeutically active agents can be packaged into EVs by either active or passive methods. Besides, P-EVs derived from a distinct species may contain therapeutic substances that boost the efficiency of the exogenous agent. However, there are several limitations regarding the application of EVs using them as a drug delivery system [125]; first, the main challenge is selecting confident and safe source cells with a high level of EVs production potential, second is selecting an operational and sensitive administration method for delivering EVs into the target sites, because EVs may be captured by the lungs and liver or deformed during loading processes, which can affect the efficiency of EVs [120, 126]. (2) EVs-based therapy is another application of EVs in medicine. As mentioned above, many researchers have shown that EVs from various plants have anticancer or regenerative properties [127, 128]. For example, EVs from different plants are cytotoxic and anti-proliferative for many tumor cells [44]. The functions of P-EVs open new opportunities for practical applications. In this context, Initial planning include the modulation of the immune system [129] or their direct participation in conveying defense proteins, RNA molecules, and antimicrobial agents [130]. This approach is helpful, however, is associated with some limitations such as selecting a distinct source with high efficiency in the isolation method. Certainly, some researchers isolated EVs from juice, whereas others isolated EVs from apoplastic fluid, including those from sunflower seeds [37, 131], which have only explored their nature, no evidence to date is existing about the origin of EVs prepared from plant mediums following squeezing. Overall, by multidisciplinary associations in cell and molecular kinetics, engineering, investigating, and medicine, we believe a hopeful future for clinical translation of P-EVs-based investigation that further studies will need to be undertaken.

## Conclusion

There is increasing interest in unravelling the composition and therapeutic potential of P-EVs. To date, several studies have reported that P-EVs are very complex, diverse structures and sizes with cellular and metabolic potential roles in plants. P-EVs are very much instrumental in delivering the natural compounds to specific cell targets, through their ability to cross bio-membranes, their biocompatibility, low toxicity, low immunogenic nature, less allergic nature and have been unable to cross placenta reducing the cross of drugs to a growing fetus. Clinical trials showed that P-EVs are promising tools for drug-delivery systems. However, the therapeutic potential of P-EVs in biomedicine is still in its infancy, largely due to complexities in extraction, purification, and other hindrances such as poor understanding of the process of biogenesis and trafficking mechanism. Further studies need to address EVs kinetics in plant cells, although existing findings are worth validating the promising therapeutic role of P-EVs and the field is worth to be explored intensely.

## Data Availability

Not applicable.

## Abbreviations

ABs: Apoptotic bodies
DCs: Dendritic cells
Dox: Doxorubicin
ECM: Extracellular matrix
ECs: Endothelial cells
ESCRT: Endosomal sorting complex required for transport
ILVs: Intraluminal vesicles
ISEV: International society for extracellular vesicles
GIPCs: Glycosyl inositol phosphoramides
GDNVs: Ginger-derived nanoparticles
GELNs: Grape-derived exosomal-like nanoparticles
GSE: Grape seed extract
miRNAs: MicroRNAs
MMPs: Matrix metalloproteinase
MSCs: Mesenchymal stem cells
MVBs: Multivesicular bodies
nSMase2: Sphingomyelinase 2
PC: Phosphatidylcholine
PE: Phosphatidylethanolamine
P-EVs: Extracellular vesicles
SNAREs: Soluble NSF attachment protein receptors
ssDNA: Single-stranded DNA
Tets: Tetraspanin
TRL: Toll-like Receptors
TSAP6: Tumor suppressor activated pathway 6
